# Machine Learning-Derived Correlations for Scale-Up and Technology Transfer of Primary Nucleation Kinetics

**DOI:** 10.1021/acs.cgd.2c00192

**Published:** 2023-01-18

**Authors:** Stephanie Yerdelen, Yihui Yang, Justin L. Quon, Charles D. Papageorgiou, Chris Mitchell, Ian Houson, Jan Sefcik, Joop H. ter Horst, Alastair J Florence, Cameron J. Brown

**Affiliations:** † EPSRC Future Continuous Manufacturing and Advanced Crystallisation Research Hub, c/o Strathclyde Institute of Pharmacy and Biomedical Sciences, University of Strathclyde, GlasgowG1 1RD, U.K.; ‡ Process Chemistry and Development, Takeda Pharmaceuticals International Company, Cambridge, Massachusetts02139, United States; § EPSRC Future Continuous Manufacturing and Advanced Crystallisation Research Hub, c/o Department of Chemical and Process Engineering, University of Strathclyde, GlasgowG1 1XQ, U.K.; ∥ Laboratoire Sciences et Méthodes Séparatives, Université de Rouen Normandie, Place Emile Blondel, Mont Saint Aignan Cedex76821, France

## Abstract

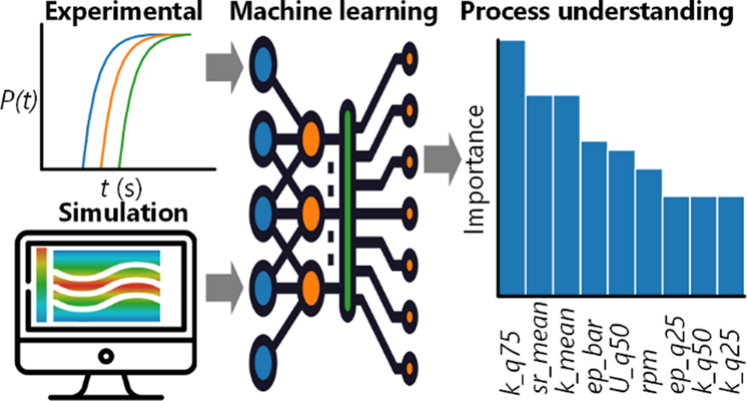

Scaling up and technology transfer of crystallization processes have been and continue to be a challenge. This is often due to the stochastic nature of primary nucleation, various scale dependencies of nucleation mechanisms, and the multitude of scale-up approaches. To better understand these dependencies, a series of isothermal induction time studies were performed across a range of vessel volumes, impeller types, and impeller speeds. From these measurements, the nucleation rate and growth time were estimated as parameters of an induction time distribution model. Then using machine learning techniques, correlations between the vessel hydrodynamic features, calculated from computational flow dynamic simulations, and nucleation kinetic parameters were analyzed. Of the 18 machine learning models trained, two models for the nucleation rate were found to have the best performance (in terms of % of predictions within experimental variance): a nonlinear random Forest model and a nonlinear gradient boosting model. For growth time, a nonlinear gradient boosting model was found to outperform the other models tested. These models were then ensembled to directly predict the probability of nucleation, at a given time, solely from hydrodynamic features with an overall root mean square error of 0.16. This work shows how machine learning approaches can be used to analyze limited datasets of induction times to provide insights into what hydrodynamic parameters should be considered in the scale-up of an unseeded crystallization process.

## Abstract 2

To better understand and predict the probability of nucleation during the scale-up of an unseeded crystallization process, a series of isothermal induction time studies were performed and machine learning techniques were used to analyze correlations between vessel hydrodynamic features and nucleation kinetic parameters.

## 1 Introduction

Solution-based crystallization processes are implemented to obtain highly pure materials for use in industries such as pharmaceuticals and fine chemicals. Critical quality attributes (CQAs), including polymorphism, crystal shape, and size distribution, are primarily affected by the nucleation event for unseeded conditions. However, to gain further control over CQAs and because of the difficulties in controlling primary nucleation, seeded crystallization processes are often used. Under such conditions, growth, secondary nucleation, and other phenomena such as agglomeration can become dominant.^1,2^

Primary nucleation is the formation of crystal nuclei from a crystal-free solution^3^ that is supersaturated (and thus thermodynamically metastable) with respect to a crystalline phase. Supersaturation can be induced by changing the solution temperature and/or composition through cooling, evaporation, mixing (e.g., in antisolvent or reactive crystallization),^4^ or a combination thereof. Homogeneous nucleation takes place in a clear supersaturated solution in the absence of any foreign material. Heterogeneous nucleation, generally regarded as the process of most practical relevance to industrial processes, involves the formation of the new solid phase as a result of the presence of interfaces present on other particles or equipment surfaces, which can reduce the energy barrier for nuclei forming on them in supersaturated solutions. In practice, this means that lower supersaturations are needed for heterogeneous nucleation. Primary nucleation is a stochastic event strongly dependent on supersaturation, and the kinetics of this process can be investigated using various approaches,^5^ including microfluidic methods under quiescent conditions and agitated solutions in vessels in different volumes.^6−10^ For industry, experimental data obtained from agitated solutions could be expected to be most relevant, as industrial processes typically involve agitated conditions. Typically, to estimate the primary nucleation kinetics of a compound from a solution, a series of isothermal induction time measurements are carried out at constant supersaturation, reactor configurations, and volume.^10^ Through application of a probabilistic stationary Poisson process, these induction time experiments can be analyzed to estimate the nucleation rate.^10^ This approach is based on the assumption that nucleation proceeds through a single nucleus mechanism (SNM), where a single nucleus is formed and grows or undergoes secondary nucleation until detection becomes possible. The assumption of an SNM has been shown to be valid for the cooling crystallization of chiral^11^ and polymorphic^12^ compounds and more recently, even in large vessel volumes.^8^

Primary nucleation kinetics have been investigated with respect to agitation^13,14^ and the effects of fluid shear.^15−18^ Agitation can have a significant effect not only on nucleation kinetics but also on the relative kinetics of nucleation of concomitant polymorphs.^19^ Unseeded crystallization operations based on primary nucleation are sometimes avoided in industry in favor of seeded crystallization to eliminate the reliance on primary nucleation, although at the cost of developing seeding strategies. In any case, understanding and controlling primary nucleation allow the boundaries of uncontrolled nucleation to be determined and avoided during experimental design, operation, and scale-up of both seeded and unseeded crystallization processes.

Crystallization events are often initially explored at milligram scale operation and allow solid-state properties of a new API to be determined. However, larger scale operations are needed to achieve a sufficiently high productivity to meet industrial demand. Scaling up/down of nucleation processes are challenges that have long been recognized,^8,20^ and the difficulties stem from the well-documented dependence of nucleation on agitation. Scale-up can be examined kinematically (e.g., impeller tip speed or impeller shear rates), dynamically (e.g., energy dissipation rate), or by geometric similarity (e.g., diameter ratio of impeller to vessel).^18^ However, limitations exist for these methods because most agitation processes rely on several mechanisms, and no single scale-up method can address them all in a realistic manner.^18^ Previous approaches used to scale-up mixing processes have been based on power input, volume averaged shear rate, and energy dissipation rate.^21−23^ These depend on the impeller shape, size, and rotational speed, as well as the vessel size and shape, affecting mixing time, suspension of particles, and contact with the high shear zones, leading to changes in primary and secondary nucleation rates.^24^ Furthermore, as the operational scale increases, variations in the mixing efficiency may lead to concentration gradients, particle suspension, and heat transfer difficulties.^25,26^ Therefore, it is of great interest to investigate potential scale-up approaches to determine their applicability to primary nucleation.

This manuscript aims to address the effects of agitation and scale on the primary nucleation kinetics using paracetamol, a compound widely studied previously and with demonstrated sensitivity to agitation.^8,27,28^ The first part evaluates the impact of impeller type and vessel hydrodynamics on induction time distributions. The second part applies data science/machine learning (ML) approaches to train models which correlate hydrodynamic parameters with primary nucleation kinetics. Model interpretability techniques are used to analyze the models to determine which hydrodynamic features are important and should be considered in process scale-up decisions.

## 2 Materials and Methods

### 2.1 Materials

All the experimental work detailed here was carried out using paracetamol obtained from Sigma-Aldrich (CAS Number: 103-90-2) with a purity greater than 98%. Isopropanol (IPA, CAS Number: 67-63-0) was sourced from VWR with a purity greater than 98%. Although the presence of impurities can be expected to affect primary nucleation, the specific impact of these was not the focus of this work.

### 2.2 Methods

#### 2.2.1 Induction Time Measurements

A series of induction time measurements were carried out across three scales: a Mettler Toledo EasyMax 102 workstation with a capacity of 100 mL, a Mettler Toledo OptiMax 1001 workstation with a capacity of 1000 mL, and a Microinnova Engineering GmBH Miniplant skid equipped with a 10 L vessel. The characteristic dimensions are given in Table 1.

**Table 1:** Dimensions of the Vessels and Impellers Investigated

scale	vessel diameter, *D* (mm)	liquid height (mm)	impeller type	impeller diameter, *d* (mm)	impeller type
100 mL	51	41.5	three-blade retreat curve (RC)	30	radial
			four-blade 45° pitch blade turbine (PBT)	25	axial
1000 mL	100	81	RC	32	radial
			PBT	45	axial
10 L	200	162	RC	110	radial

A Mettler Toledo FBRM probe (G400 series) was used with icFBRM V4.3 incorporated in the iControl software V5.4. The solution was heated to at least 10 °C above the theoretical solubility temperature, gathered from the literature,^29,30^ where it remained for at least 30 min to ensure complete dissolution of all crystals (validated by FBRM total counts showing <10 #/s). Using a cooling rate of 3 °C/min, the solution was cooled to 15 °C. While the solution was supersaturated for a period of time before the experimentally relevant temperature was reached, the FBRM probe was used to confirm that nucleation had not occurred during the cooling stage. The moment 15 °C was reached was taken as the beginning of the experiment. The maximum achievable cooling rate for the 10 L vessel was determined to be 0.9 °C/min. Therefore, for the induction time experiments on the 10 L scale, a different cooling rate was applied. Mettler-Toledo iControl software was programmed to repeat these heating and cooling cycles by triggering the next experiment at the point when the FBRM detected a minimum of 1000 crystals. All induction time measurements were carried out at 15 °C, corresponding to a supersaturation ratio, *S* = *c*/c*, of 1.6, where *c* = 155.81 g/kg solvent and c* = 97.38 g/kg solvent.^29^ These conditions were chosen to allow comparison to the previous literature^8^ and are expected to have induction times within a reasonable timescale. A condenser was used to prevent solvent loss. The solution was stirred vigorously after each cycle to ensure that any material that may have been deposited on the walls of the vessel was removed. Each induction time measurement was repeated a minimum of five times using the same solution. The solution was not filtered to duplicate industrial conditions. The type of impellers studied and their respective dimensions can be seen in Table 1.

#### 2.2.2 Distribution of Induction Times

In the model proposed by Jiang and ter Horst^10^ it is assumed that, due to the stochastic nature of nucleation, the probability of forming a particular number of crystals over a time interval follows a Poisson distribution. The induction time, *t*, that is observed experimentally is the time it takes until particles are detected in the supersaturated solution. This can be further broken down into a primary nucleation event at time, *t*_n_, and the growth time, *t*_g_, which is the time for the crystal nuclei to form a detectable suspension. Therefore, the proportion, *P*(*t*), of observations made by time *t* is defined in eq 1:^10^
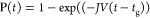
1Here, *J* is the rate of nucleation per unit volume, and *V* is the volume of the solution. Experimentally, for a number of induction times, *t_i_*, *i* = 1, 2, ..., *M*, ordered from shortest to longest, the observed cumulative probability, *P*(*t*), is then:
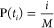
2

The average induction time *t_M_®* is defined through
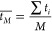
3

Note that in practice samples are not given an infinite time to nucleate. As a result, when samples do not nucleate within a given time, the average induction time can be misleading as it does not consider these “missing” induction times with values larger than the given observation time. Therefore, in these cases, it is better to take the median induction time. To estimate *J* and *t*_g_, data in the form of eq 2 can be fitted directly to eq 1 using a least-squares method. For this fitting to be accurate and to ensure that the probability distribution of nucleation events is well sampled, it has been recommended that a relatively large number (80+) of induction times should be collected.^9,10,16,31,32^ However, due to the experimental scales used in this study, this number of induction times would take a significant resource in terms of time and material to obtain. Therefore, to improve the accuracy of the estimation of *J* and *t*_g_ from fewer induction time measurements, additional statistical techniques were employed to enhance the measured data: bootstrapping, maximum likelihood estimation (MLE) and a combination of both. In all cases induction time observation values were used directly with no preprocessing.

#### 2.2.3 Direct Fit

The observed induction times were sorted in ascending order, and the observed cumulative probability was calculated according to eq 2. Eq 1 was then fitted to the observed induction times, *t*_i_, and cumulative probability, *P*(*t*_i_) using a nonlinear least-squares method to estimate parameters *J* and *t*_g_. This minimizes the sum of squared differences between the cumulative probabilities and those calculated from eq 1. Based on initial fitting, values for *J* and *t*_g_ were constrained from 0.01 to 20 #/(m^3^ s) and 3000 to 30,000 s, respectively.

#### 2.2.4 Bootstrapping

Bootstrapping the observed induction times through random sampling allows one to mimic the sampling process, thereby allowing for the estimation of the sample distribution. The experimental data were randomly resampled 1000 times (with replacement so that a given data point can be reselected) for each experimental condition. Thus, 1000 new sets of observed induction times were generated which mimic the original experimentally observed induction time distribution. For each new resample, the cumulative probability was calculated as per eq 2 and fitted, again using a least-squares method, to eq 1. The mean and standard deviation values of *J* and *t*_g_ of these 1000 fits were then calculated. This bootstrapping approach has been shown to be valuable in estimating standard deviations in other nucleation systems.^15,33^

#### 2.2.5 Maximum Likelihood Estimation

MLE is especially useful for nonlinear modeling with nonuniformly distributed data and assumes that for the underlying distribution, the observed data are the most probable. As a result, the MLE parameters return a unique stationary point that is the global maximum. MLE has been shown to be a powerful methodology for the estimation of the nucleation characteristics of gas hydrates.^34^ The likelihood for one sample can be written as
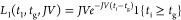
4

This means that the likelihood for *M* samples is:
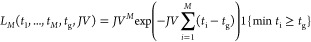
5

Taking into account two cases, we note that if *t*_g_ > min *t*_i_, then 1{ min *t*_i_ ≥ *t*_g_} = 0 and hence *L_M_* (*t*_1_, ..., *t_M_*, t_g_, *JV*) = 0. On the other hand, if *t*_g_ ≤ min *t*_i_, then 1{ min *t_i_* ≥ *t*_g_} = 1, and:
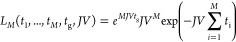
6which is an increasing function of *t*_g_. Therefore, for any fixed values of *JV*, the maximum likelihood estimator for *t*_g_ is:
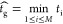
7

This estimation for *t*_g_ has been used previously^10,35^ and makes the implicit assumption that a sufficient number of induction times have been measured, such that one of them represents an instance where the time at which a single nucleus has formed is equal to 0 s.^36^ For this value of *t*_g_, the logarithmic likelihood can now be optimized:
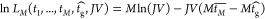
8
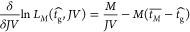
9

Setting the first derivative to zero then yields the following:
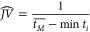
10

#### 2.2.6 Maximum Likelihood Estimation plus Bootstrapping

For the resampled datasets used to bootstrap the direct fitting of data to eq 1, the MLE parameters, eqs 7 and 10, were also calculated.

#### 2.2.7 Synthetic Induction Times Generation

To compare the approaches for estimation of induction time distribution parameters without underlying experimental variations, only by stochastic variations, a range of synthetic induction times were generated: The distribution parameters *J*, *V*, and *t*_g_ were arbitrary (although are comparable to the parameters of the real 10 L dataset) set to 0.1 #/m^3^ s, 0.01 m^3^, and 10,000 s, respectively. For *M* observations (where *M* = 5, 10, 20, 40, or 80), a random number generator was used to generate values of *P*(*t*) within the range [0, 1]. The synthetic induction time, *t*, was then calculated using these *P*(*t*) values by rearranging eq 1.

#### 2.2.8 Computational Fluid Dynamic (CFD) Simulations

CFD models for all vessel/impeller configurations in Table 1 were built using COMSOL Multiphysics 5.4. A 3D frozen rotor model was built using Turbulent Flow, *k*–ε, physics within the package. Free tetrahedral meshes were generated using physics-controlled settings for the element size, ensuring that the average element quality was consistent between the different models. Hydrodynamic parameters generated as a result of the simulations are given in Table 2. For each parameter, there will be a distribution of values across the volume of the vessel. Therefore, to capture this, quantiles (25, 50, and 75%) of the distributions were recorded.

**Table 2:** Hydrodynamic Parameters Extracted from CFD Simulations

parameter	term	unit
shear rate, mean	sr_mean	1/s
shear rate, 25% quantile	sr_q25	1/s
shear rate, 50% quantile (median)	sr_q50	1/s
shear rate, 75% quantile	sr_q75	1/s
turbulent dissipation rate, mean	ep_mean	m^2^/s^3^
turbulent dissipation rate, 25% quantile	ep_q25	m^2^/s^3^
turbulent dissipation rate, 50% quantile (median)	ep_q50	m^2^/s^3^
turbulent dissipation rate, 75% quantile	ep_q75	m^2^/s^3^
turbulent kinetic energy, mean	k_mean	m^2^/s^2^
turbulent kinetic energy, 25% quantile	k_q25	m^2^/s^2^
turbulent kinetic energy, 50% quantile (median)	k_q50	m^2^/s^2^
turbulent kinetic energy, 75% quantile	k_q75	m^2^/s^2^
velocity magnitude, mean	U_mean	m/s
velocity magnitude, 25% quantile	U_q25	m/s
velocity magnitude, 50% quantile (median)	U_q50	m/s
velocity magnitude, 75% quantile	U_q75	m/s
axial velocity, mean	axial_mean	m/s
radial velocity, mean	radial_mean	m/s
power draw	power_draw	W/m^3^

#### 2.2.9 Other Hydrodynamic Measures

In addition to the hydrodynamic parameters captured in Table 2, basic parameters were also calculated, which are commonly used in the scale-up of stirred vessels. These include the Reynolds number, *Re*, impeller tip speed, *u*_tip_, and specific power input, ε̅:
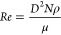
11
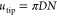
12
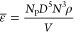
13where *D* = impeller diameter (m), *N* = impeller speed (1/s), ρ = fluid density (kg/m^3^), μ = fluid viscosity (Ns/m^2^), *N*_p_ = power number (1.3 for pitched blade and 1.07 for retreat curve), and *V* = volume (m^3^).

#### 2.2.10 Regression Analysis

To understand the relationships between the induction time parameters and hydrodynamic parameters, a regression analysis workflow was developed in Orange: Data Mining Toolbox in Python v3.27.1.^37^ Vessel volume, impeller speed, and the hydrodynamic parameters were imported as numerical features, whereas the impeller type was imported as a categorical feature. The type was also imported as meta-data to easier track the vessel but was not used as a feature for regression analysis. The induction time distribution parameters for each configuration, *J* and *t*_g_, were imported as numerical targets. The preprocessing of the data consisted of the taking the log of distribution parameter, *J*, normalizing the numerical features by standardization (μ = 0, σ^2^ = 1) and converting the categorical features to hot features, i.e., conversion of each categorical value into a new numerical feature and assign a binary value of 1 or 0. For regression models, linear (linear, ridge, and LASSO) and nonlinear (Random Forest, gradient boosting, and *k*-nearest neighbors (*k*NN)) algorithms were selected and trained against the same dataset. To evaluate the performance of each model, the data set was divided into two subdatasets: training and validation (*n* = 14), and testing (*n* = 3). Within the training and validation subdataset, a *leave one out* approach was taken due to the small dataset size. The hyperparameters of each model, i.e., parameters that control the learning process of the model, were independently tuned for log(*J*) and *t*_g_ to minimize the root mean square deviation (RSME) between prediction and observation and are given for each model in the ESI.

#### 2.2.11 Overall Data Analysis Scheme

**Figure 1:**
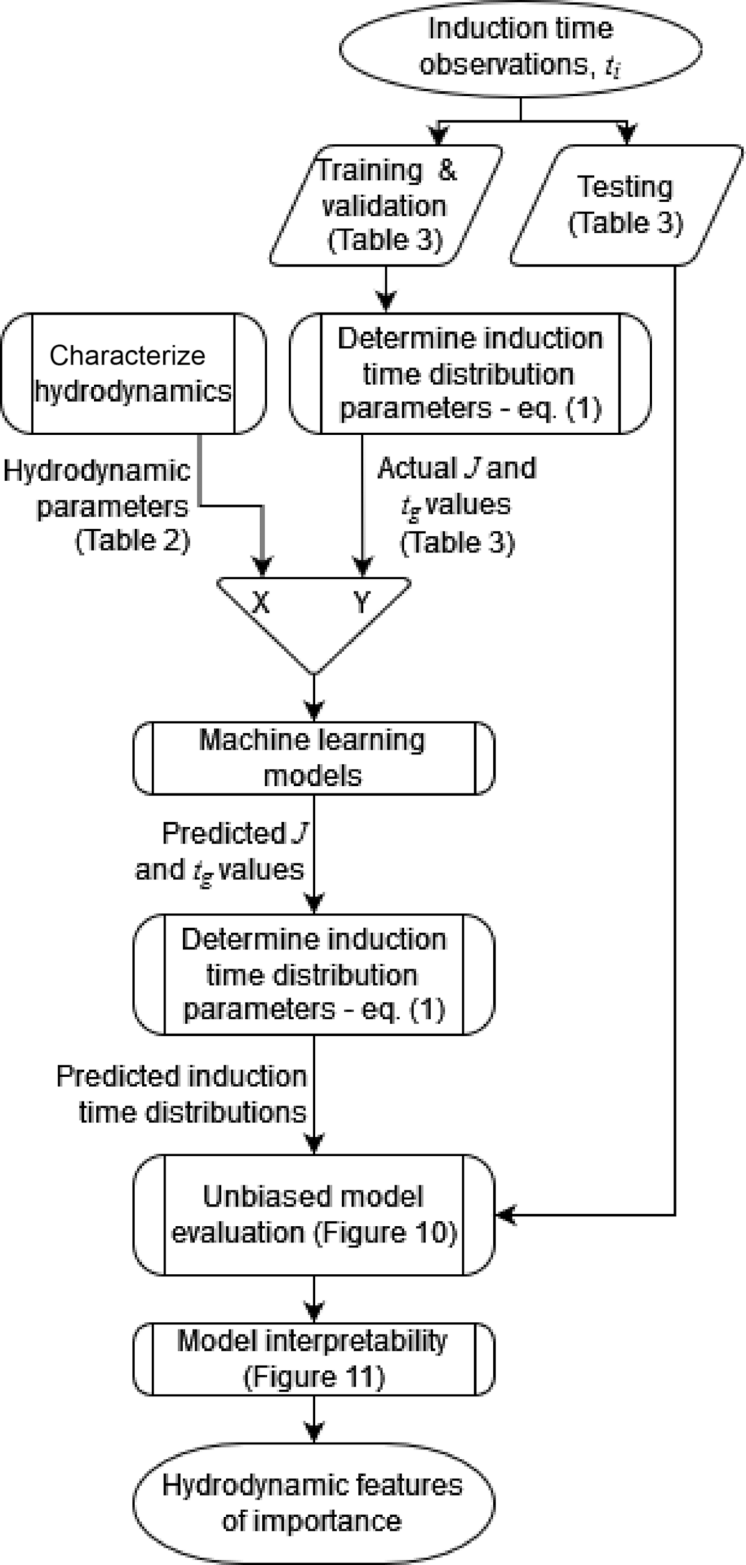
Overall data analysis scheme detailed in this article.

Taking the above methods into account, Figure 1 shows how these were incorporated into an overall data analysis scheme. Initially, induction time observations (Section 2.2.1) were made over a range of vessel and impeller configurations (Table 1). These were separated into observations for ML model training and validation, and testing (Table 3). Observations for model training and validation were used to estimate induction time distribution parameters *J* and *t*_g_ (Section 2.2.2) via eq 1. In parallel, the hydrodynamic parameters for each vessel and impeller configuration were calculated by CFD (Sections 2.2.8 and 2.2.9). A series of ML models were trained and evaluated which correlate *J* and *t*_g_ with the hydrodynamic parameters. Once trained, these models were used to predict *J* and *t*_g_ and the resulting induction time distribution for the vessel and impeller configurations assigned to testing. These predicted induction time distributions were compared with the actual induction time observations of the testing dataset to provide an unbiased evaluation of the model fit. Finally, model interpretability techniques were applied to the best performing models to interpret which hydrodynamic features have the greatest effect on *J* and *t*_g_.

**Table 3:** Fitted Induction Time Distribution Parameters to Two Significant Figures, to Data in Figure 3, RSME between Fits and Observed Cumulative Probability, and Use of Data in the Development of ML Models

vessel	volume (mL)	impeller type	impeller speed (rpm)	number of induction times (*M*)	*J* (#/m^3^ s)	*t*_g_ (s)	RMSE	use in ML model development
EasyMax	85	RC	150	5	1.000	25,000	0.16	training and validation
EasyMax	85	RC	225	8	1.600	21,000	0.07	training and validation
EasyMax	85	RC	315	14	2.800	14,000	0.15	training and validation
EasyMax	85	RC	353	8	1.600	12,000	0.09	training and validation
EasyMax	85	RC	365	6	1.800	9800	0.15	training and validation
EasyMax	85	RC	383	7	2.800	11,000	0.13	training and validation
EasyMax	85	RC	450	8	0.770	9200	0.14	training and validation
EasyMax	85	PBT	400	11	1.500	4200	0.11	training and validation
EasyMax	85	PBT	330	8	1.300	11,000	0.11	training and validation
EasyMax	85	PBT	267	9	0.480	3600	0.13	training and validation
OptiMax	630	RC	300	6	0.180	7000	0.15	training and validation
OptiMax	630	PBT	250	6	0.150	14,000	0.13	training and validation
OptiMax	630	PBT	310	7	0.220	3300	0.08	training and validation
Miniplant	9300	RC	165	7	0.034	13,000	0.11	training and validation
EasyMax	85	RC	250	9	2.300	18,000	0.07	testing
OptiMax	630	RC	220	7	0.120	6500	0.12	testing
Miniplant	9300	RC	300	5	0.084	8600	0.17	testing

## 3 Results and Discussion

### 3.1 Comparison of Approaches for the Estimation of Distribution Parameters

**Figure 2:**
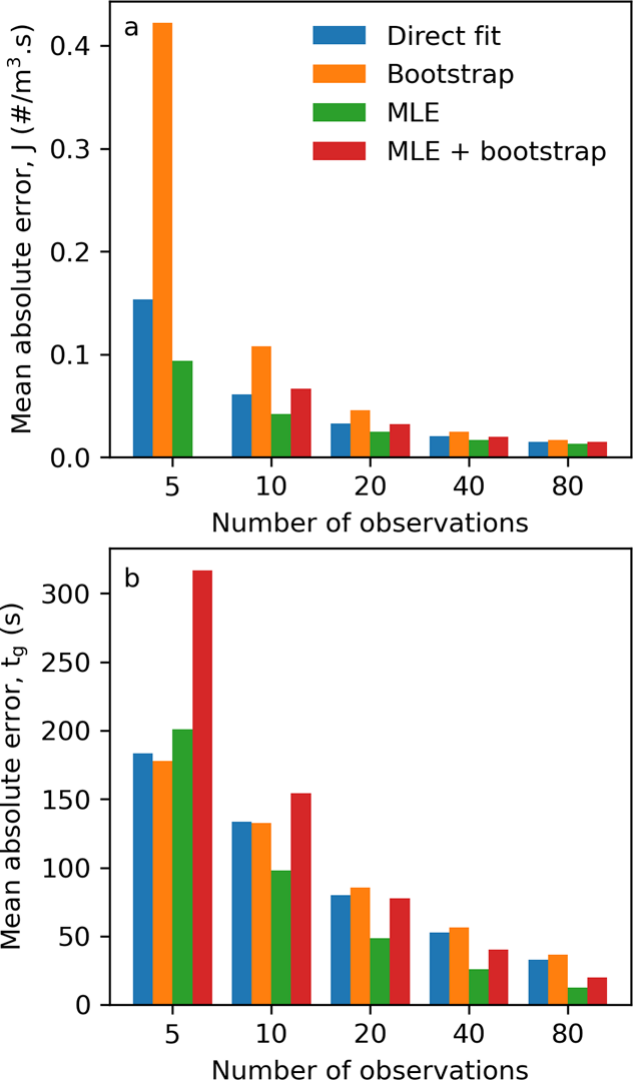
Comparison of mean absolute error of different Poisson distribution parameters: direct fitting, bootstrapping, MLE, and MLE + bootstrapping for (a) nucleation rate, *J* = 0.1 #/m^3^ s, and (b) growth time, *t*_g_ = 10,000 s. The MAE for *J* from the MLE + bootstrapping method with five observations is not shown, as it consistently produces an MAE value order of magnitude greater than the other methods.

To compare the methodologies for the estimation of the distribution parameters, 1000 synthetic induction time datasets were generated for *M* = 5, 10, 20, 40, and 80 observations (5000 datasets in total). For every dataset, the mean absolute error (MAE) between the estimated *J* and *t*_g_ and the ground truth values (*J* = 0.1 #/m^3^ s and *t*_g_ = 10,000 s, the results for other values of *J* are shown in the ESI) of each approach (direct fitting, bootstrapping, MLE, and MLE + bootstrapping) was calculated and averaged over 1000 datasets. These average MAEs as a function of the number of observations are shown in Figure 2.

Unsurprisingly, there is a noticeable reduction in MAE as the number of observations increases for both the estimation of *J* (Figure 2a) and *t*_g_ (Figure 2b). Taking into account the average MAEs for the estimated *J* and *t*_g_ values across the 1000 synthetic datasets versus the ground truth values, the MLE approach produced the smallest errors for *J* and *t*_g_ across the majority of observation number scenarios. Previous work investigating the uncertainty from induction time measurements in small volumes^36^ compared different approaches to estimating *t*_g_: minimum induction time (i.e., MLE), fitting to data or calculated from growth kinetics. In that study it was found, assuming that nucleation follows a Poisson distribution, that setting *t*_g_ to the shortest induction time (i.e., the same approach as that used in the MLE approach) is acceptable when using 80 or more induction time observations. The results of the work presented here would suggest that this approach is also acceptable when using sparse, e.g., <20, observations. In contrast, in the same study^36^ they demonstrated that for *J* the most accurate way to estimate it is by minimizing the sum of squared differences between the model and experiments, that is, a direct fitting approach, while the results here suggest that for sparse data (<20 observations), the MLE approach is more accurate for the range of *J* and *t*_g_ values relevant to this study. Therefore, to estimate the Poisson distribution parameters from the sparse experimental data, the MLE approach was used throughout, unless otherwise stated. Furthermore, the errors derived here have been assumed to be constant when applied to real data in subsequent sections.

### 3.2 Induction Time Distributions

**Figure 3:**
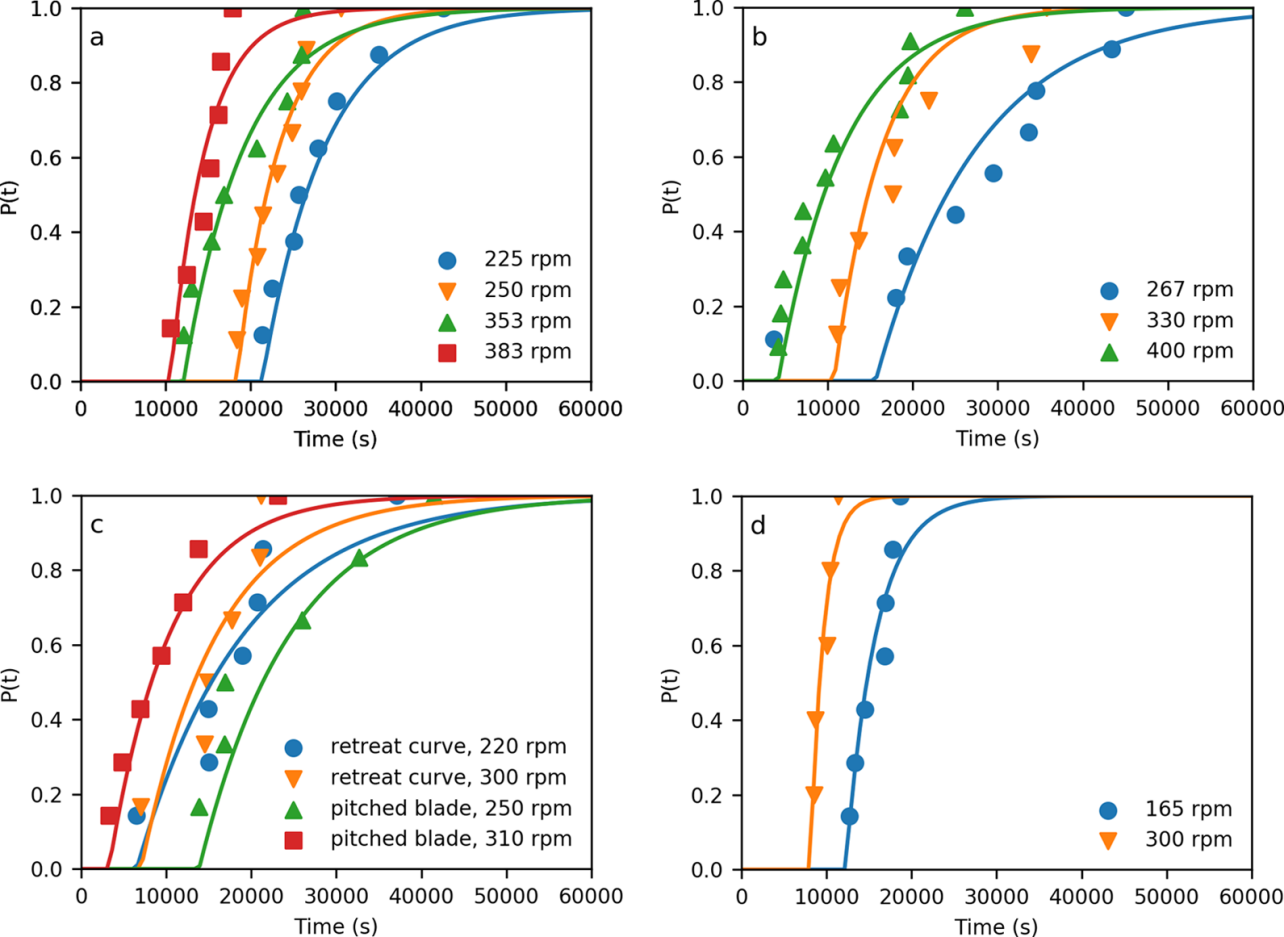
Probability distributions *P*(*t*) of the induction times, *t*_i_, for *S* = 1.6 measured in crystallization volumes of (a) 85 mL with a retreat curve impeller (not all conditions are shown for clarity), (b) 85 mL with a pitched blade impeller, (c) 630 mL with retreat curve and pitched blade impellers, and (d) 9300 mL with a retreat curve impeller. Lines are fits to eq 1 with parameters estimated through MLE.

The probability distributions of induction times for each vessel, impeller, and rpm are fitted to eq 1 and shown in Figure 3, and parameters are tabulated in Table 3. The MLE approach and eqs 7 and 10 were used to estimate the nucleation rate *J* and growth time, *t*_g_. The root mean square error (RMSE) between the fit and observed cumulative probability calculated from eq 2 is also given in Table 3.

For the smallest volume, 85 mL, with a retreat curve impeller, Figure 3a, increasing the impeller speed from 225 to 383 rpm clearly shifts the distribution to the left, i.e., reducing *t*_g_. A similar trend is seen for the pitch blade turbine impeller, also at 85 mL, Figure 3b, where increasing the impeller speed from 267 to 400 rpm shifts the distribution to the left. However, for a smaller change in speed a greater shift in the distribution is observed. d*t_M_®*/d*N* (change in average induction time for change in impeller speed) is 40 s/rpm for the retreat curve impeller and 121 s/rpm for the pitched blade. This suggests a different dependency on impeller speed with impeller type and is likely to be related to different hydrodynamics and/or secondary nucleation. The impact of the impeller type can also be seen for a 630 mL volume, Figure 3c, where for a retreat curve impeller the distributions at different stirrer speeds are almost overlapping, while for a pitch blade impeller, they show an obvious shift with the pitch blade impeller speed. For the largest volume experiments, Figure 3d, the induction times are still relatively large, suggesting that nucleation occurs via an SNM.^20^

**Figure 4:**
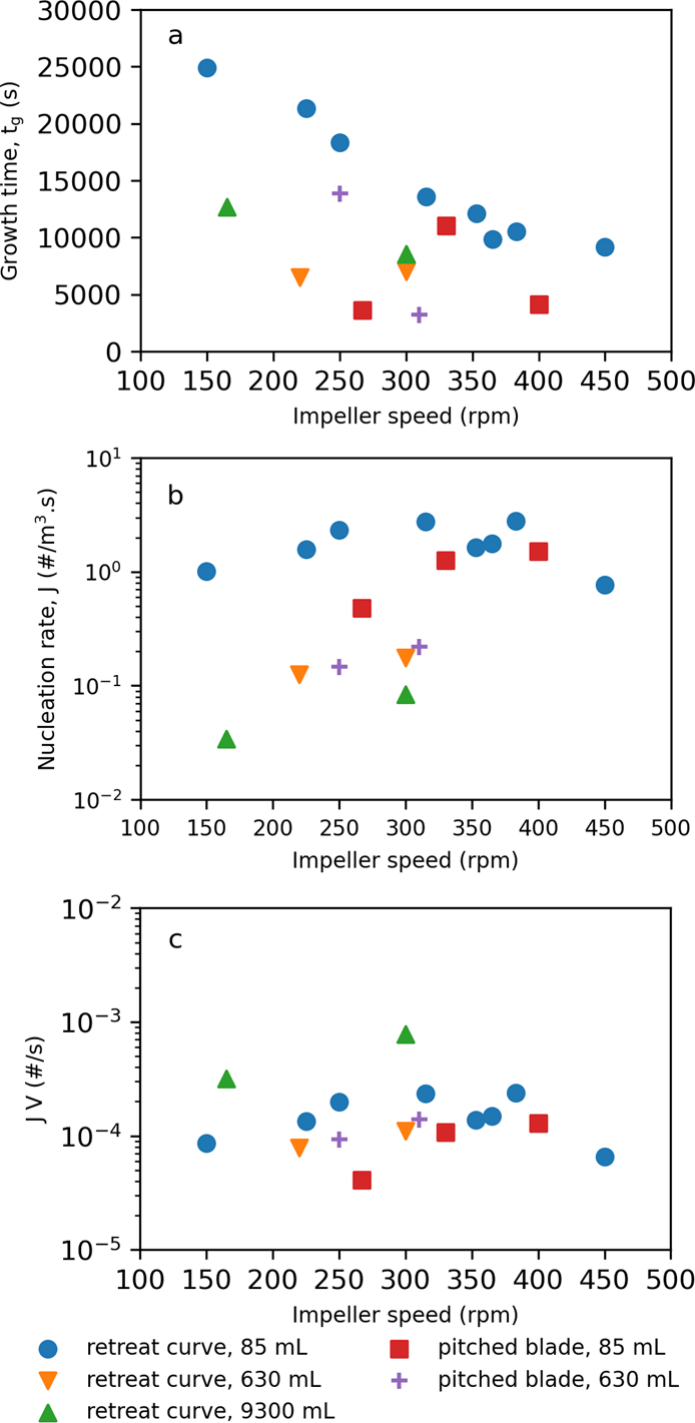
Induction time distribution parameters, (a) *t*_g_, (b) *J*, and (c) *JV*, as a function of impeller speed for a range of vessel volumes and impeller type.

Figure 4a shows that *t*_g_ in for the EasyMax (85 mL) with a retreat curve impeller decreases with increasing impeller speed. The Optimax (630 mL) with a pitched blade and Miniplant (9300 mL) with a retreat curve potentially show the same trend but are limited to two datapoints. As *t*_g_ is the time for the number and size of particles to reach a detection threshold and that the growth of paracetamol has previously been shown to be limited by the surface integration of molecules onto the crystals^38^ (i.e., growth rate is agitation independent), then this trend must be due to enhanced secondary nucleation with agitation. This could be due to two mechanisms: (1) crystals only need to grow to a smaller threshold size for secondary nucleation to occur, or (2) there are more nuclei produced per unit time for a parent crystal of a given size. Two configurations do not show this trend with impeller speed, EasyMax (85 mL) with a pitched blade impeller and OptiMax (630 mL) with a retreat curve impeller, which show a maximum or no change, respectively. This was observed consistently, regardless of which approach was used to estimate the distribution parameters. Investigating EasyMax (85 mL) with the pitched blade configuration (Figure 4a red squares) further with the MLE plus bootstrapping approach reveals that for the data point at 267 rpm, *t*_g_ has a significantly higher standard deviation (±7394 vs 1309 and 572 s) than the other data points for this series (and is, in fact, the highest of all configurations). With this into account, an inversely proportional relationship, in line with the rest of the data, between impeller speed and *t*_g_ would be feasible. A similar investigation into the OptiMax (630 mL) with a retreat curve impeller configuration (Figure 4a orange downward triangles) also revealed noticeably larger standard deviations for *t*_g_ for both data points (220 and 300 rpm) compared to the rest of the datasets. Along with the EasyMax data point previously discussed, these three data points have the three highest standard deviations for all the configurations. In retrospect, this would suggest that these configurations would have benefited from further experimental points to allow better estimation of the distribution parameters.

Figure 4b shows two major observations for *J*: (1) within a specific configuration, *J* possibly increases with increasing impeller speed (although not conclusively) and (2) values for *J* cluster at different magnitudes (10^–2^, 10^–1^, and 10^0^ #/m^3^ s) according to the configuration volume. With regard to observation 1, the link between nucleation rate, *J*, and impeller speed has been well established and shown for a number of other systems and configurations.^14−16,39^ This is due to increasing impeller speed leading to increased shear, both in laminar and turbulent regimes, resulting in increased primary nucleation rates.^15^ However, the exact mechanisms of this at the molecular level are an open scientific question and not conclusively answered. Furthermore, in the data presented here, as some of the configurations only have two data points with impeller speed, a conclusive trend cannot be determined. For observation 2, there is approximately an order of magnitude change in the values for *J* with an order of magnitude change in volume, i.e., nucleation rate at constant supersaturation is a function of volume. Similar observations were made by Dela Cruz et al.,^40^ in unstirred confined small volumes where nucleation rates were observed to go through a maximum with increasing volume. Forsyth et al.^41^ also observed an increase in nucleation rates as a function of vessel specific surface areas in a laminar regime. This suggests that nucleation is not likely to occur in the whole volume equally. Plotting *JV* in Figure 4c reveals indeed for the smaller volumes (85 and 630 mL), and *JV* remains relatively constant 0.13 × 10^–3^ ± 0.047 × 10^–3^ #/s across both volumes and impeller type. However, *JV* for the largest volume (9300 mL) lies significantly outside this range, suggesting perhaps a different nucleation mechanism for the larger volume. This is not unusual, as it is often assumed that secondary nucleation in small volume solution proceeds through mechanical attrition where a single crystal breaks into fragments after collision with the impeller.^20^ However, in larger volumes, crystal/impeller collisions are less frequent^42^ and secondary nucleation follows a breeding mechanism,^43^ for which there is some experimental evidence for paracetamol.^44^

In summary, investigating the induction time distributions for two different impeller types (retreat curve and pitched blade) over a range of impeller speeds and vessel volumes reveals that while the distribution parameters (*J* and *t*_g_) follow the same general trends between impeller types, there are differences in how responsive these parameters are due to changes in impeller speed. This is expected as the pitched blade turbine will generate a mixture of axial and radial flow, while the retreat curve will generate predominantly axial flow.^45^ Therefore, the following section will investigate the specific detailed hydrodynamic parameters that correlate with the induction time distribution parameters.

### 3.3 Correlation of Hydrodynamics and Nucleation Rate, *J*

Full details of the hydrodynamic parameters, Table 2, from the CFD simulations and additional calculated parameters, eqs 11–13, for each configuration are given in the ESI.

As part of the comparison of approaches for the estimation of distribution parameters, the error associated with the estimation of *J* was calculated as a function of observations of the number of induction time, *M* (Figure 2a). These can be considered equivalent to the experimental error in the measurement of *J*. With such an error in the experimental data, the common model performance metrics, *R*^2^ and RMSE, can be less reliable.^46^ Therefore, two additional metrics were defined: % of predictions within the *J* ± error for *M* = 5, (% ± *M* = 5) and % of predictions within the *J* ± error for *M* = 20, (% ± *M* = 20). The former reflecting maximum accuracy of a model with the fewest observations and the latter reflecting the accuracy of model observations greater than performed. The performance metrics for all the models developed are given in Table 4.

**Table 4:** Summary of Performance Metrics for All Models for the Prediction of *J* with *R*^2^ Values of NaN Representing Poor Fits between Model and Data, for Which the Data Are Better Represented by Assuming the Mean Value

			training dataset				testing dataset	
model no.	type	feature(s)	RMSE (#/m^3^ s)	*R*^2^	% ± M = 5	% ± M = 20	RMSE (#/m^3^ s)	*R*^2^
J1	univariate	*Re*	1.294	NaN	50.0	42.9	2.882	NaN
J2	univariate	Utip	1.224	0.048	14.3	14.3	1.815	NaN
J3	univariate	ε̅	1.382	NaN	7.1	0.0	2.403	NaN
J4	univariate	radial_mean	0.929	0.452	14.3	14.3	2.253	NaN
J5	ridge	reduced	5.219	NaN	14.3	14.3	1.699	NaN
J6	LASSO	reduced	1.047	0.304	14.3	14.3	1.560	NaN
J7	random Forest	reduced	0.709	0.681	42.9	42.9	0.353	0.943
J8	kNN	reduced	1.204	0.078	14.3	0.0	1.806	NaN
J9	gradient boosting	reduced	0.672	0.713	28.6	28.6	0.340	0.947

#### 3.3.1 Univariate Benchmark Models

**Figure 5:**
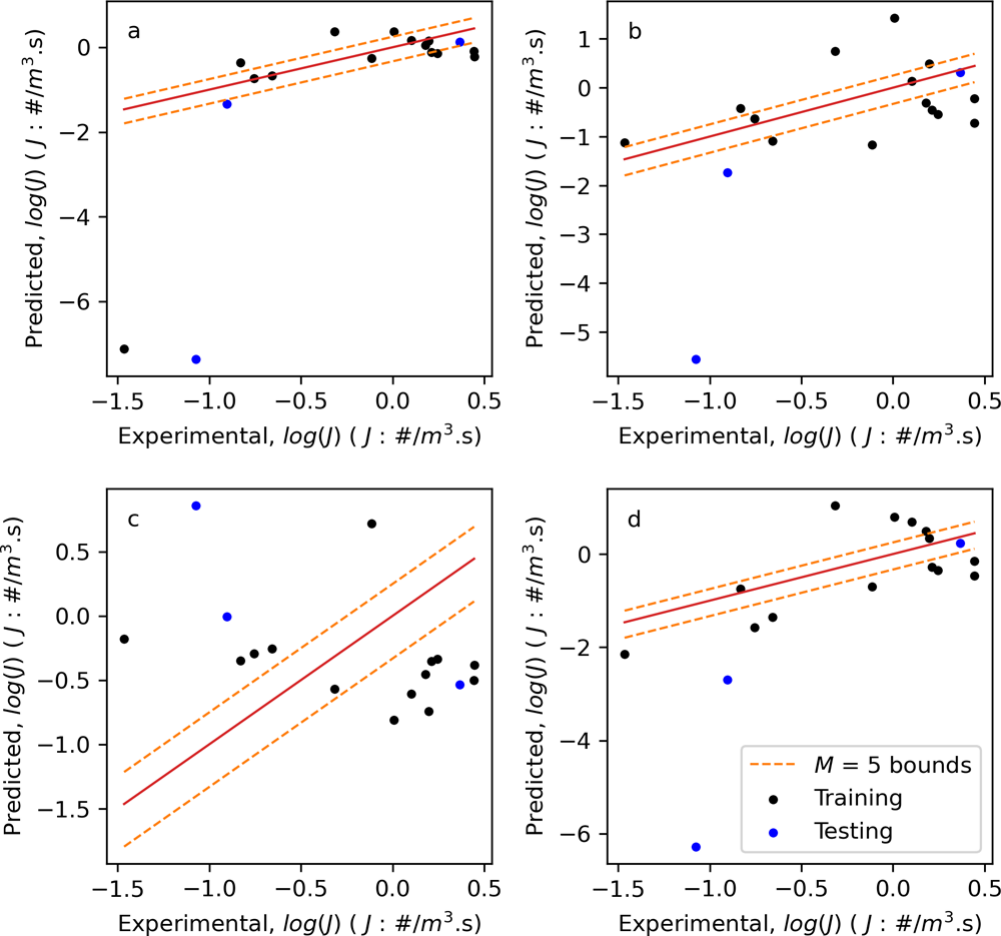
Plots of predicted vs experimental log(*J*) for univariate models using (a) *Re*, (b) *u*_tip_, (c) ε̅, and (d) radial_mean. Bounds (orange dashed lines) represent the experimental error for five observations of induction time.

To set a benchmark performance for the prediction of *J*, univariate linear regression models between *J* and common scale-up parameters (*Re*, *u*_tip_, ε̅ ) were developed. Additionally, Pearson correlation analysis of the hydrodynamic parameters and *J* showed that the mean radial velocity had the greatest correlation with *J*. The plots of predicted vs experimental *J* are shown in Figure 5. These graphs and the performance metrics in Table 4 indicate that the performance of these models is mixed with a maximum of the 50% of predictions within the largest experimental error limits for the *Re* model (J1) on the training dataset. However, Figure 5a shows that there are two observations (one each from the training and testing datasets) where the predicted *J* is significantly different from the actual value. Model J4 (mean radial velocity) is the next best performing with 14.3% of observations within experimental error bounds, but the lowest RMSE and highest *R*^2^ for these univariate models on the training dataset. However, Figure 5d again shows that these predictions are far from ideal. Furthermore, the RMSE values for all these models on the testing dataset show them to be noticeably higher than those of the training dataset and none return a valid *R*^2^ value. Given the complicated flow patterns expected across a range of vessel geometries, it is perhaps unsurprising that a single parameter cannot accurately capture such a relationship. Nevertheless, scaling up chemical processes based on a single parameter is common practice,^47^ and these results provide benchmark performance metrics.

#### 3.3.2 Reduced Features and Multivariate Regression Models

**Figure 6:**
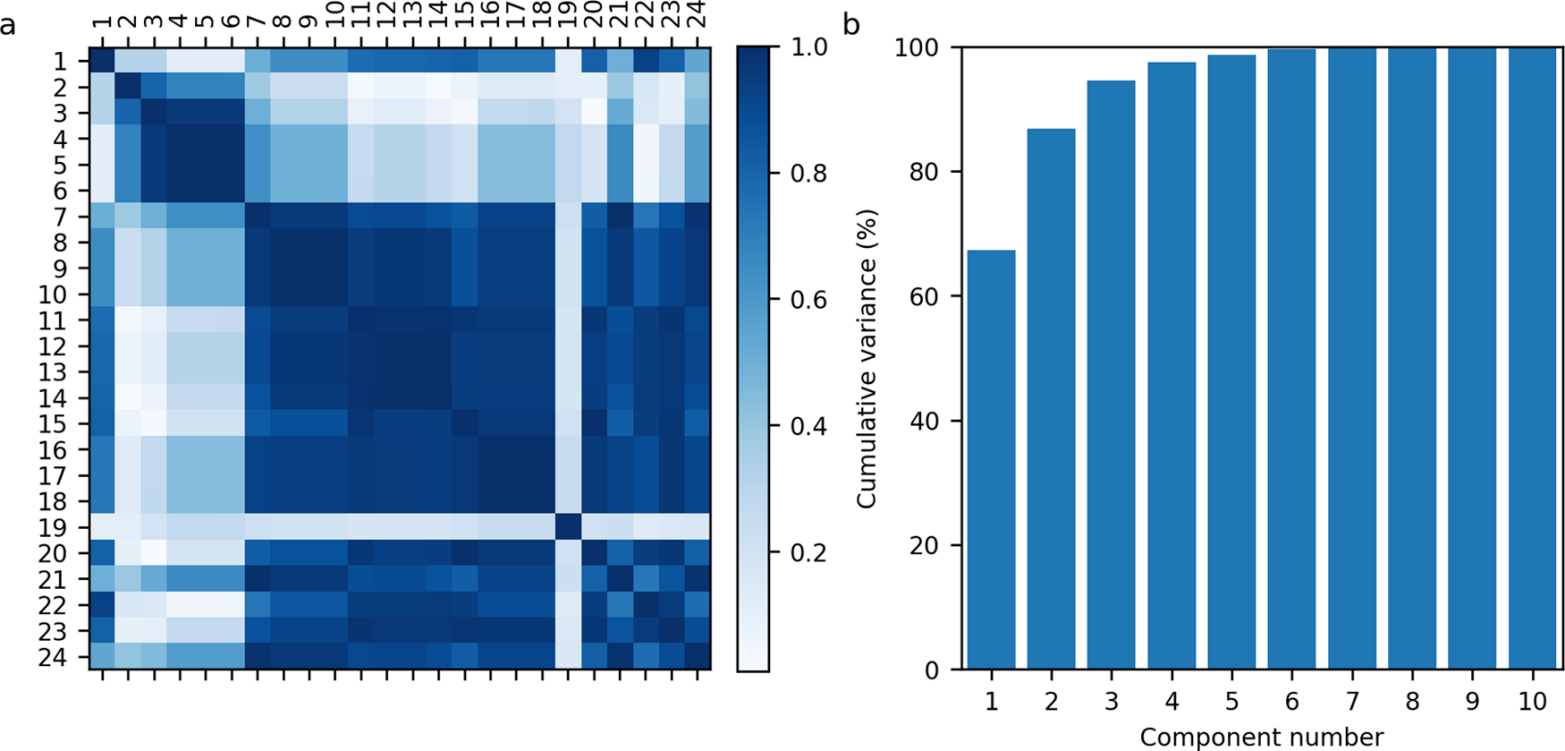
(a) Feature Pearson correlation analysis with darker shading indicating a higher correlation and (b) feature principal component analysis.

Before generating multivariate regression models, it is common to remove highly correlated features to prevent multicollinearity. Calculating the Pearson correlation coefficients for all 24 features, Figure 6a, reveals that many of the features are highly correlated. This is perhaps expected as many of the features represent the quantiles of a hydrodynamic parameter distribution. Therefore, if the 50% quantile increased, we would also assume that the 25 and 75% quantiles would also increase. Using an acceptable correlation threshold of ≤0.95, the features were reduced to 6: volume, impeller speed, sr_mean, ep_mean, axial_mean, and impeller type. This reduced feature set was termed “reduced” in Table 4. Similarly, principal component analysis of the 24 features, Figure 6b, indicates that >6 components accounts for the variation in the features.

**Figure 7:**
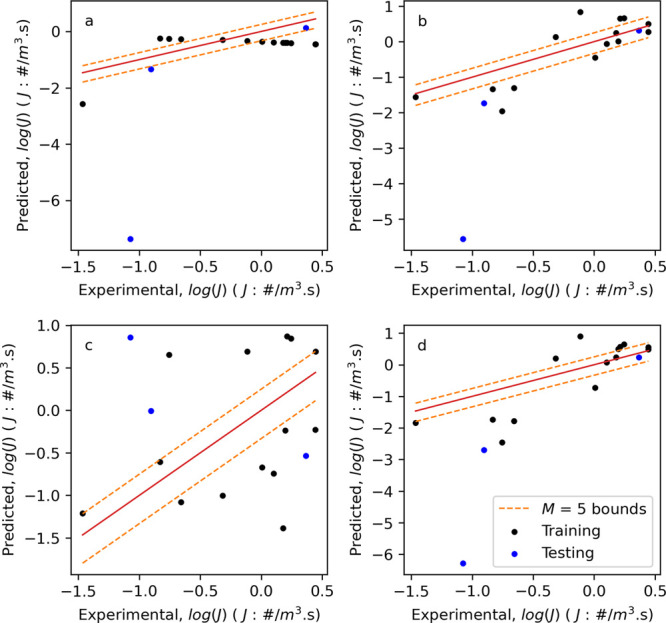
Plots of predicted vs experimental log(*J*) for (a) model J6—LASSO linear regression, (b) model J7—Random Forest, (c) model J8—kNN, and (d) model J9—Gradient Boosting. Bounds (orange dashed lines) represent the experimental error for five observations of induction time.

Using this reduced feature set, both ridge and LASSO linear regression models were fitted along with nonlinear Random Forest, kNN and Gradient Boosting models. Their performance metrics are given in Table 4, and the plots of the predicted vs experiment *J* are shown in Figure 7. By comparing traditional performance metrics (RMSE and *R*^2^) on the training dataset, these models provide an improvement compared to the benchmark models (with the exception of the ridge model, J5), with a reduction in RMSE (0.929 vs 0.672 for model J4 and J9, respectively) and an increase in *R*^2^ (0.452 vs 0.713 for model J4 and J9, respectively). When using the performance metrics based on the experimental error, these models perform comparable to the benchmark models with 42.9% of predictions within experimental error for 5 and 20 observations for model J7 (Random Forest) and 28.6% for model J9 (Gradient Boosting). Additionally, in the test dataset these two models (J7 and J9) perform well, with the lowest RMSE values of all models and *R*^2^ values greater than 0.9. Further analysis of the performance of models J7 and J9 will be discussed later when predicting induction time distributions.

### 3.4 Correlation of Hydrodynamics and Growth Time, *t*_g_

As mentioned previously, MAE values (Figure 2b) were used as surrogates for experimental measurement of *t*_g_. However, these errors are relatively small compared to the values for *t*_g_. As a result, the determination of additional performance metrics (% ± *M* = 5) and (% ± *M* = 20) result in very tight limits with none of the developed models achieving greater than 0%. Therefore, RMSE was used as the only metric for the comparison of model performance and is shown in Table 5 for all models.

**Table 5:** Summary of Performance Metrics for All Models for the Prediction of *t*_g_ with *R*^2^ Values of NaN Representing Poor Fits between the Model and Data, for Which the Data Are Better Represented by Assuming the Mean Value

			training		testing	
model no.	type	feature(s)	RMSE (s)	*R*^2^	RMSE (s)	*R*^2^
T1	constant value		5976	NaN	5166	NaN
T2	constant values		5165	0.25	5665	NaN
T3	univariate	rpm	5844	0.044	5757	NaN
T4	univariate	*u*_tip_	7182	NaN	8735	NaN
T5	ridge	reduced	33,615	NaN	5126	0.015
T6	LASSO	reduced	11,291	NaN	5820	NaN
T7	random Forest	reduced	6112	NaN	4868	0.112
T8	kNN	reduced	5585	0.127	3443	0.556
T9	gradient boosting	reduced	5652	0.106	3532	0.532

#### 3.4.1 Constant Value Benchmark Models

**Figure 8:**
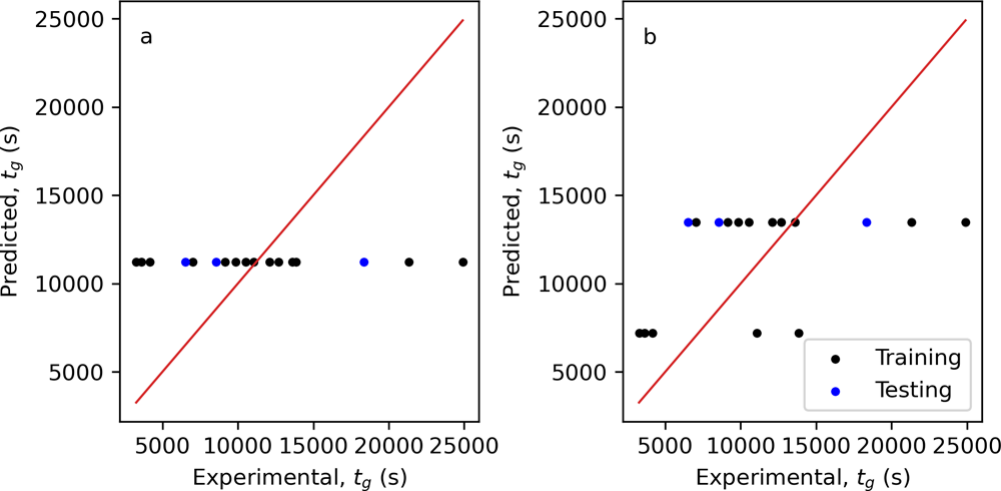
Plots of predicted vs experimental *t*_g_ for (a) model T1—single mean value across all observations and (b) model T2—mean values separated by the impeller type.

To set a benchmark performance of the prediction of *t*_g_, two models, T1 and T2, assuming a single constant value for *t*_g_ were developed. For model T1, it is assumed that *t*_g_ is the mean value of *t*_g_ across training observations, i.e., *t*_g_ = 11,226 s. Model T2 takes the same approach, but classifies the observations into those for the pitch blade and retreat curve impellers and calculates the mean value of *t*_g_ for each class (Figure 8). Therefore, *t*_g_ is calculated as shown in eq 14:
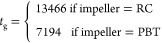
14

The performance of these models is interesting, particularly T2 as it has the lowest RMSE for the training dataset. As shown above (Figure 4a), *t*_g_ in general decreases with impeller speed with separate trends based on volume and impeller type. Therefore, it would be expected for a model to consider these features as highly important features. As described above, *t*_g_. is a function of both growth and secondary nucleation, with growth assumed to be independent of agitation. As a result, changes in *t*_g_ could be attributed to different secondary nucleation rates. Furthermore, changes in the secondary nucleation rate would be expected to be captured by the hydrodynamic features, Table 2. However, the performance of model T2 suggests that changes in the secondary nucleation rate could be consolidated into two classes based on the impeller type. However, unusually this would mean that the secondary nucleation rate would be independent of any hydrodynamic feature, e.g., rpm, axial velocity, shear rate, etc.

#### 3.4.2 Reduced Features and Multivariate Regression Models

**Figure 9:**
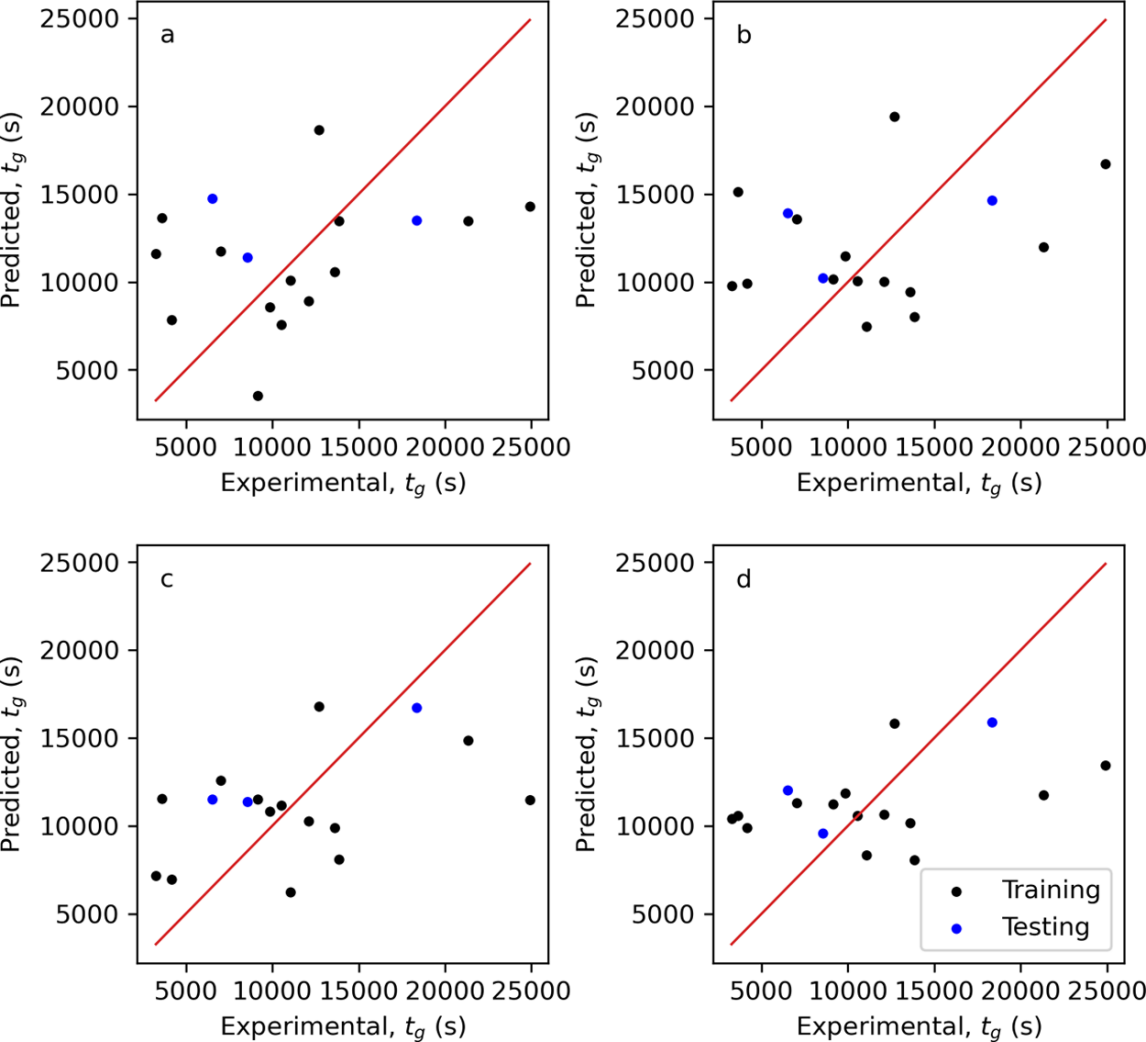
Plots of predicted vs experimental *t*_g_ for (a) model T3—univariate using impeller speed, (b) model T7—Random Forest, (c) model T8—kNN, and (d) model T9—Gradient Boosting.

To investigate whether there are correlations between *t*_g_ and any hydrodynamic feature, univariate linear regression models between *t*_g_ and common scaling parameters (impeller speed and tip speed. Both parameters were indicated by Pearson correlation to have a strong or medium correlation, respectively) were developed. Additionally, using the reduced feature set, described in Section 3.3.2, both ridge and LASSO linear regression models were fitted along with nonlinear random Forest, kNN, and gradient boosting models. Their performance metrics are given in Table 5 and the plots of predicted vs experiment *t*_g_ are shown in Figure 9.

Of the univariate models, model T3 (using impeller speed, rpm), Figure 9a, shows the best performance with RMSE values on the training and testing datasets comparable to those of the constant value model by impeller type (model T2), 5844 vs 5165 s (training) and 5757 vs 5665 s (testing), respectively. Interestingly, the ridge and LASSO linear regression models (T5 and T6, respectively) show poor performance on the training dataset with RMSE values an order of magnitude higher than the benchmark models (33,615 vs 5165 s for T5), but performance comparable to the benchmark models on the testing dataset. Extending this further to the nonlinear models, T8 (kNN, Figure 9c) and T9 (gradient boosting, Figure 9d), again shows performance on the training dataset comparable to the benchmark models (5585 vs 5165 s for T8), but also shows increased performance on the testing dataset (3443 vs 5665 s for T8). This increase in performance suggests that changes in *t*_g_ attributed to different secondary nucleation rates can be captured when hydrodynamic features are considered. Further analysis of the performance of these T8 and T9 will be discussed later when predicting the induction time distributions.

### 3.5 Prediction of Induction Time Distributions

**Figure 10:**
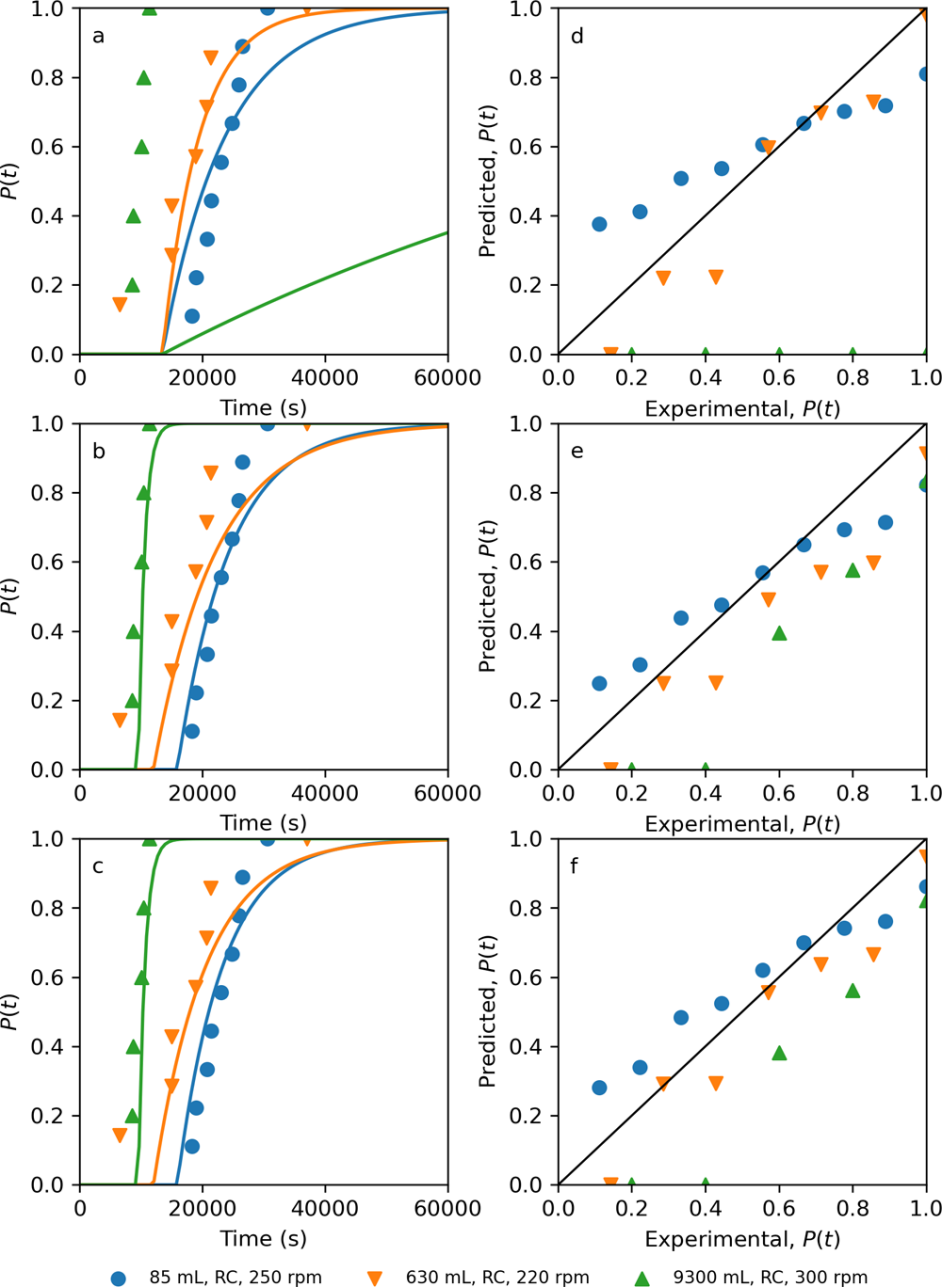
Probability distributions *P*(*t*) of the measured induction times *t*_i_. The lines are predicted probability distributions from the ensemble models (a) J2 + T2, (b) J7 + T9, and (c) J9 + T9. Plots of predicted vs experimental *P*(*t*) ensemble models (d) J2 + T2, € J7 + T9, and (f) J9 + T9.

To further test the performance of the trained models, the best performing models for predicting *J* and *t*_g_ on the testing dataset (J7, J9, T8, and T9) were taken to form four model ensembles, which along with eq 1 could be used to predict induction time distributions, solely from the hydrodynamic features. As a comparative benchmark, an ensemble of J2 and T2 was also included to represent the case where no CFD or machine learning model development was performed. Therefore, the prediction of the induction time distributions relied solely on common scale-up parameters, i.e., tip speed for *J* and a constant value for *t*_g_. To compare performance, the aggregated RMSE and *R*^2^ across all the predicted induction time distributions were calculated for the testing vessel configurations in Table 3 and are shown in Table 6 and comparison of the predicted induction time distribution to the experimental results in Figure 10 for the best performing ensembles and no CFD or machine learning benchmark.

**Table 6:** Summary of Performance Metrics for Model Ensembles for the Prediction of Induction Time Distributions

model ensemble	RMSE	*R*^2^
J2 + T2 (no CFD or ML benchmark)	0.35	0.22
J7 + T8	0.34	0.32
J7 + T9	0.17	0.80
J9 + T8	0.33	0.30
J9 + T9	0.16	0.76

From Table 6 and Figure 10a,d, it is clear that the benchmark model ensemble (J2 + T2) using no CFD or machine learning models has particular poor performance at the larger Miniplant scale (9300 mL). This is reflected in the highest RMSE and lowest *R*^2^ of all ensembles in Table 6, and in Figure 10a it predicts that for the Miniplant configuration the probability of nucleation occurs at times greater than 13,496 s, whereas in reality the whole probability distribution lies less than this value. Suggesting that this ensemble is not generalizable to wider configurations. Of the other model ensembles, using the *t*_g_ model T9 outperforms the ensembles using *t*_g_ model T8, as the RMSE decreases (0.34 vs 0.17) and the *R*^2^ increases (0.32 vs 0.8). However, which *J* model is performing better is a little less clear. Ensemble J9 + T9 has the lower RMSE compared to J7 + T9 (0.16 vs 0.17), but it also has a lower *R*^2^ value (0.76 vs 0.80). These differences are subtle but can be observed in the predicted induction time distributions in Figure 10a,b and are most noticeable for the 85 and 630 mL scale configurations. The 630 mL scale specifically shows a very different trend between the induction time observations and predicted distribution, particularly at the low and high induction times. However, this difference is also observed between the MLE fitted distributions and observed cumulative distribution in Figure 3c (blue circles), suggesting that these observations could be outliers. Overall though, in comparison to the experimentally fitted induction time distributions using the MLE approach, Table 3, the aggregated RMSE for ensembles J7 + T9 and to J9 + T9 are of a comparable value: 0.07, 0.12, and 0.17, for each testing configuration, respectively, vs 0.17 and 0.16 for J7 + T9 and to J9 + T9, respectively. This result demonstrates that regardless of which of these two ensembles is chosen, both demonstrate that the trained machine learning models for *J* (Section 3.3.2) and *t*_g_ (Section 3.4.1) can be used to predict the probability, *P*(*t*), of nucleation for a given time, *t*, with an error comparable to inherent fitting errors, from only the hydrodynamic features.

### 3.6 Model Interpretability

**Figure 11:**
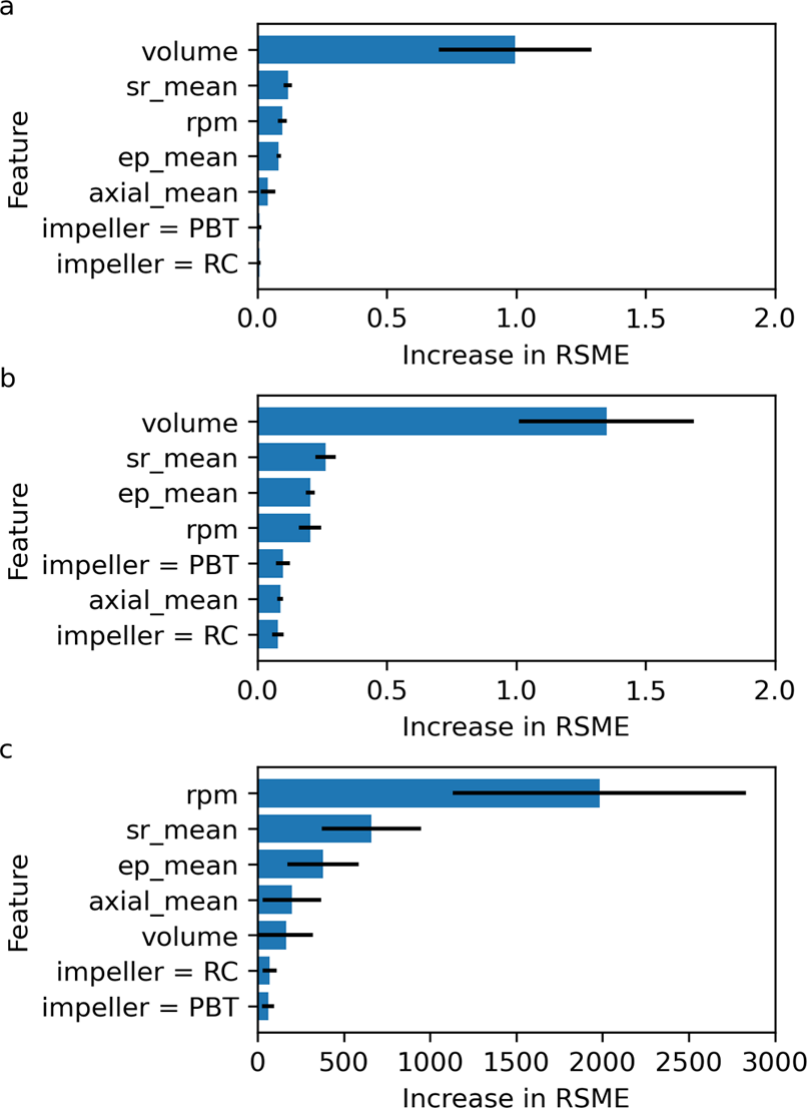
. Global effect of features for models (a) J7, (b) J9, and (c) T9.

To probe further into these model ensembles, SHAP (Shapley Additive exPlanations) interpretations can be used to compute the feature importance for the models J7, J9, and T9. The absolute SHAP values for the features in these models are shown in Figure 11, and further plots detailing the directional impact of the features are shown in the ESI. For models J7 and J9, Figure 11a,b, the vessel volume has the largest effect on the prediction of *J*, this is followed by sr_mean, ep_mean, and rpm (although the exact ordering of these does change between J7 and J9). Similarly, for model T9, Figure 11c, the features that have the largest effect on the prediction of *t*_g_ are rpm, sr_mean, and ep_mean. While sr_mean and ep_mean are common important features for the prediction of *J* and *t*_g_, their directional effect is different. For the prediction of *J* in models J7 and J9, high values of sr_mean and ep_mean have a positive effect on *J*, i.e., increasing sr_mean and/or ep_mean increases *J*. On the contrary, for the prediction of *t*_g_ in model T9, high values of sr_mean have a positive effect on *t*_g_, but high values of ep_mean have a negative effect on *t*_g_. A potential outcome of this is that, if trying to optimize hydrodynamic features to maintain an induction time distribution across scales/technologies, a compromise will have to be made in the consistency of *J* or *t*_g_, i.e., optimizing for one could be at the detriment of the other.

It is also worth noting that while SHAP identifies which features are important to the model, they do not necessarily indicate a causal link.^48^ However, the importance of these hydrodynamic features is consistent with previous studies.^21−23^

## 4 Conclusions

We have shown that, for the calculation of induction time parameters, the nucleation rate *J* and the growth time *t*_g_, from limited experimental observations, <10 per measurement series, an MLE approach has the lowest mean absolute error relative to the true induction time distribution. The MLE approach was then used to calculate *J* and *t*_g_ from induction time observations across a range of vessel scales, impeller types, and impeller speeds at a fixed supersaturation. A range of regression models were then trained with the correlation of *J* and *t*_g_ to hydrodynamic parameters calculated from CFD simulations of vessel configurations. For *J*, a Random Forest (J7) or gradient boosting (J9) model was found to have the best performance (RMSE = 0.353 and 0.340 #/m^3^ s, respectively) when tested on an external testing dataset. For *t*_g_, a gradient boosting model (T9) was found to perform best (RMSE = 3532 s) when tested on an external test dataset. These models were then ensembled to allow for the prediction of the probability of nucleation at a given time from only hydrodynamic parameters with an overall RMSE of 0.16. Furthermore, these models were found to be more generalized compared to those using traditional scale-up parameters. Model interpretability techniques also highlighted that particular note should be taken of the mean shear rate and turbulent dissipation rates between the small-scale experiments and at-scale equipment to maintain a consistent induction time distribution.

The outcome of this work shows how data science/traditional machine learning approaches can be used to analyze even limited datasets of induction times (131 observations) to derive insights into what hydrodynamic parameters might be important to consider in an unseeded crystallization process, thus reducing risks and improving scale-up and technology transfer, resulting in reduced environmental impact through less waste.

## Supporting Information Available

Results of the comparison of approaches for estimating distribution parameters for other values of *J*, calculated hydrodynamic features for each vessel configuration, trained model hyperparameters, and model interpretability summaries for models J7, J9, and T9 (PDF)

## Notes

All data underpinning this publication are openly available from the University of Strathclyde KnowledgeBase at https://doi.org/10.15129/5a7908ad-2cdf-492f-a3bf-d1e96c8d80d6: observed induction times and calculated *J* and *t*_g_ parameters using the MLE approach, predicted *J* and *t*_g_ values for each trained machine learning model and associated model performance metrics, and predicted induction time distributions on testing observations and associated model performance metrics.
