# Supplementary material for: Machine Learning-Derived Correlations for Scale-Up and Technology Transfer of Primary Nucleation Kinetics
Source: Cryst Growth Des. 2023 Jan 18;23(2):681–93. doi: 10.1021/acs.cgd.2c00192 (PMC9896482; doi:10.1021/acs.cgd.2c00192)
Supplement: Supplementary file 1 — cg2c00192_si_001.pdf [file cg2c00192_si_001.pdf]

Supporting Information

Machine learning derived correlations for scale-up and technology transfer of primary nucleation kinetics

*Stephanie Yerdelen[1], Yihui Yang[2], Justin L. Quon[2], Charles D. Papageorgiou[2], Chris Mitchell[2], Ian Houson[1], Jan Sefcik[3], Joop H. ter Horst[1,4], Alastair J Florence[1], Cameron J. Brown[1]**

[1] EPSRC Future Continuous Manufacturing and Advanced Crystallisation Research Hub, c/o Strathclyde Institute of Pharmacy and Biomedical Sciences, University of Strathclyde, Glasgow, United Kingdom.

[2] Process Chemistry and Development, Takeda Pharmaceuticals International Company, Cambridge, Massachusetts, United States

[3] EPSRC Future Continuous Manufacturing and Advanced Crystallisation Research Hub, c/o Department of Chemical and Process Engineering, University of Strathclyde, Glasgow, United Kingdom

[4] Laboratoire Sciences et Méthodes Séparatives, Université de Rouen Normandie, Place Emile Blondel, 76821 Mont Saint Aignan Cedex, France

* Corresponding author

E-mail address: cameron.brown.100@strath.ac.uk

Comparison of approaches for estimation of distribution parameters

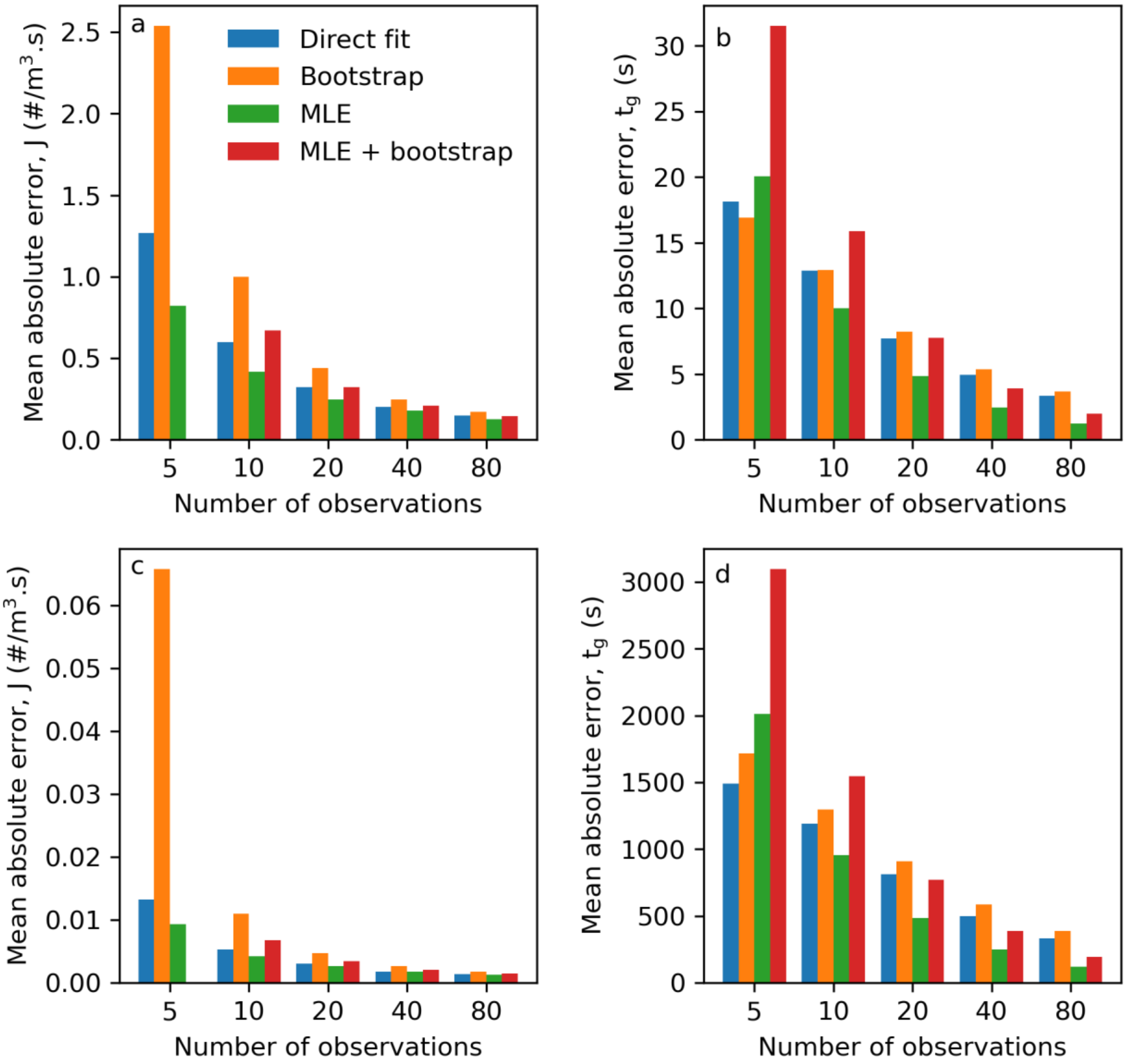

Figure 1. Mean absolute error comparison of different Poisson distribution parameter estimation methods: direct fitting, bootstrapping, MLE and MLE + bootstrapping for a) J at J = 1 #/m^3.s, t_g = 10,000 s, b) t_g at J = 1 #/m^3.s, t_g = 10,000 s, c) J at J = 0.001 #/m^3.s, t_g = 10,000 s and d) t_g at J = 0.01 #/m^3.s, t_g = 10,000 s.

Hydrodynamic parameters for vessel configurations

Vessel	Volume (mL)	Impeller type	Impeller speed (rpm)	sr_mean (s^{-1})	sr_q25 (s^{-1})	sr_q50 (s^{-1})	sr_q75 (s^{-1})	ep_mean (m^2/m^3)	ep_q25 (m^2/m^3)	ep_q50 (m^2/m^3)	ep_q75 (m^2/m^3)
EasyMax	85	RC	150	12	19	37	55	0.008	0.022	0.045	0.067
EasyMax	85	RC	225	19	31	61	91	0.025	0.080	0.159	0.238
EasyMax	85	RC	250	21	35	69	103	0.033	0.108	0.216	0.324
EasyMax	85	RC	315	27	45	90	134	0.063	0.206	0.411	0.616
EasyMax	85	RC	353	31	51	102	153	0.086	0.277	0.553	0.829
EasyMax	85	RC	365	32	53	106	159	0.095	0.300	0.600	0.900
EasyMax	85	RC	383	34	57	113	169	0.108	0.336	0.671	1.006
EasyMax	85	RC	450	40	68	136	203	0.169	0.408	0.815	1.222
EasyMax	85	PBT	400	21	25	49	73	0.027	0.043	0.086	0.128
EasyMax	85	PBT	330	17	20	40	60	0.016	0.026	0.051	0.077
EasyMax	85	PBT	267	13	17	33	48	0.009	0.015	0.029	0.044
OptiMax	630	RC	220	21	33	65	97	0.062	0.181	0.360	0.540
OptiMax	630	RC	300	28	45	90	134	0.149	0.362	0.723	1.084
OptiMax	630	PBT	250	21	29	58	86	0.081	0.200	0.399	0.599
OptiMax	630	PBT	310	17	24	47	70	0.044	0.118	0.235	0.352
Miniplant	9300	RC	165	11	21	42	62	0.053	0.224	0.448	0.672
Miniplant	9300	RC	300	21	43	86	128	0.292	1.211	2.417	3.624

Vessel	Volume (mL)	Impeller type	Impeller speed (rpm)	k_mean (m^2/s^2)	k_q25 (m^2/s^2)	k_q50 (m^2/s^2)	k_q75 (m^2/s^2)	U_mean (m/s)	U_q25 (m/s)	U_q50 (m/s)	U_q75 (m/s)
EasyMax	85	RC	150	0.0013	0.0015	0.0030	0.0044	0.06	0.05	0.10	0.15
EasyMax	85	RC	225	0.0025	0.0031	0.0061	0.0091	0.10	0.08	0.15	0.23
EasyMax	85	RC	250	0.0029	0.0037	0.0074	0.0110	0.11	0.09	0.17	0.26
EasyMax	85	RC	315	0.0043	0.0057	0.0113	0.0169	0.14	0.11	0.22	0.33
EasyMax	85	RC	353	0.0051	0.0070	0.0139	0.0208	0.16	0.12	0.24	0.36
EasyMax	85	RC	365	0.0054	0.0074	0.0148	0.0222	0.17	0.13	0.25	0.37
EasyMax	85	RC	383	0.0058	0.0081	0.0162	0.0242	0.18	0.13	0.26	0.39
EasyMax	85	RC	450	0.0076	0.0110	0.0219	0.0219	0.21	0.15	0.31	0.46
EasyMax	85	PBT	400	0.0027	0.0020	0.0040	0.0060	0.09	0.08	0.15	0.23
EasyMax	85	PBT	330	0.0020	0.0015	0.0029	0.0043	0.07	0.06	0.12	0.19
EasyMax	85	PBT	267	0.0014	0.0011	0.0021	0.0031	0.06	0.05	0.10	0.15
OptiMax	630	RC	220	0.0073	0.0068	0.0135	0.0202	0.29	0.14	0.28	0.41
OptiMax	630	RC	300	0.0129	0.0120	0.0240	0.0359	0.40	0.18	0.37	0.55
OptiMax	630	PBT	250	0.0083	0.0083	0.0165	0.0247	0.27	0.14	0.27	0.40
OptiMax	630	PBT	310	0.0055	0.0055	0.0110	0.0164	0.21	0.11	0.22	0.33
Miniplant	9300	RC	165	0.0107	0.0138	0.0276	0.0413	0.41	0.17	0.34	0.51
Miniplant	9300	RC	300	0.0331	0.0431	0.0860	0.1290	0.77	0.31	0.62	0.93

Vessel	Volume (mL)	Impeller type	Impeller speed (rpm)	axial_mean (m/s)	radial_mean (m/s)	Power_draw (W/m^3)	*Re*	u_{tip} (m/s)	$\bar{\varepsilon}$ (W/m^3)
EasyMax	85	RC	150	-6.08E-05	5.17E-02	8.4	630	0.24	3.8
EasyMax	85	RC	225	-8.79E-05	8.49E-02	26.3	945	0.35	12.8
EasyMax	85	RC	250	-9.68E-05	9.60E-02	35.4	1049	0.39	17.5
EasyMax	85	RC	315	-1.07E-04	1.26E-01	67.8	1322	0.49	35.1
EasyMax	85	RC	353	-1.10E-04	1.43E-01	93.6	1482	0.55	49.3
EasyMax	85	RC	365	-1.10E-04	1.49E-01	102.8	1532	0.57	54.5
EasyMax	85	RC	383	-1.10E-04	1.57E-01	117.8	1608	0.60	63.0
EasyMax	85	RC	450	-1.08E-04	1.88E-01	185.9	1889	0.71	102.2
EasyMax	85	PBT	400	-3.03E-04	7.13E-02	26.0	1166	0.52	35.0
EasyMax	85	PBT	330	-2.47E-01	5.68E-02	15.1	962	0.43	19.7
EasyMax	85	PBT	267	-1.69E-04	4.44E-02	8.3	778	0.35	10.4
OptiMax	630	RC	220	1.98E-06	2.85E-01	65.4	3072	0.63	34.8
OptiMax	630	RC	300	2.40E-06	3.97E-01	154.5	4188	0.86	83.2
OptiMax	630	PBT	250	-1.73E-04	2.62E-01	81.5	2897	0.73	40.9
OptiMax	630	PBT	310	-1.35E-04	2.08E-01	43.9	2337	0.59	21.4
Miniplant	9300	RC	165	-3.78E-06	4.02E-01	56.3	9383	0.96	32.8
Miniplant	9300	RC	300	-2.14E-06	7.62E-01	311.2	17060	1.74	190.5

Model hyperparameters

Table 1. Model hyperparameters for the prediction of *J*.

Model no.	Type	Feature(s)	Hyperparameter	Value
J1	Univariate	Re	-	-
J2	Univariate	utip	-	-
J3	Univariate	$\bar{\varepsilon}$	-	-
J4	Univariate	radial_mean	-	-
J5	Ridge	Reduced	Regularization strength, α	1000
J6	LASSO	Reduced	Regularization strength, α	0.8
J7	Random Forest	Reduced	Number of trees	500
J8	kNN	Reduced	Number of neighbors	3
J9	Gradient Boosting	Reduced	Number of trees, learning rate	500, 0.010

Table 2. Model hyperparameters for the prediction of t_g.

Model no.	Type	Feature(s)	Hyperparameter	Value
T1	Constant value	-	-	-
T2	Constant values	-	-	-
T3	Univariate	rpm	-	-
T4	Univariate	u_{tip}	-	-
T5	Ridge	Reduced	Regularization strength, α	1000
T6	LASSO	Reduced	Regularization strength, α	1000
T7	Random Forest	Reduced	Number of trees	500
T8	kNN	Reduced	Number of neighbors	4
T9	Gradient Boosting	Reduced	Number of trees, learning rate	500, 0.001

Model interpretability

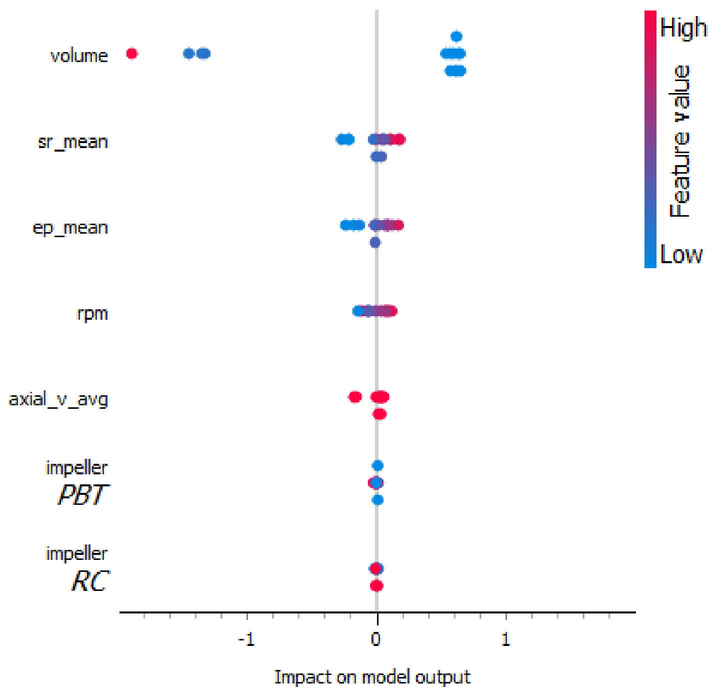

Figure 2. Model interpretability summary plot for model J7

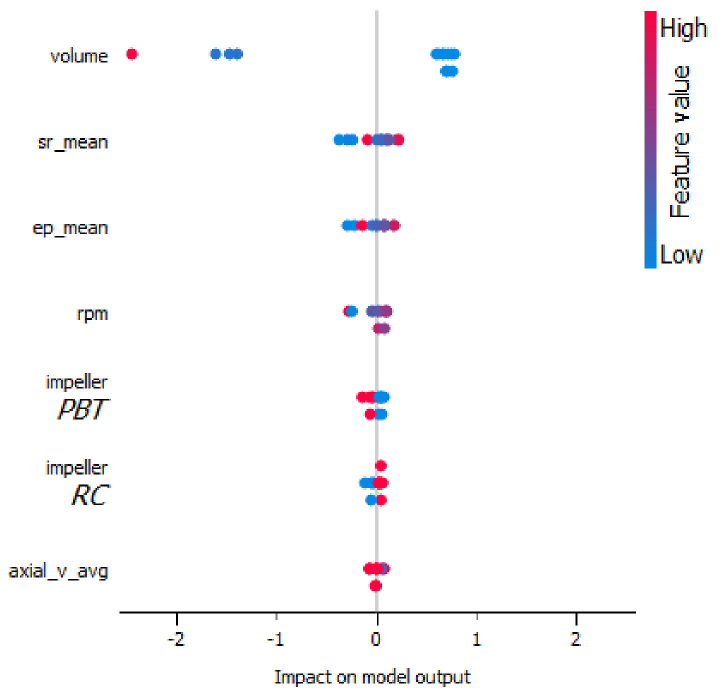

Figure 3. Model interpretability summary plot for model J9

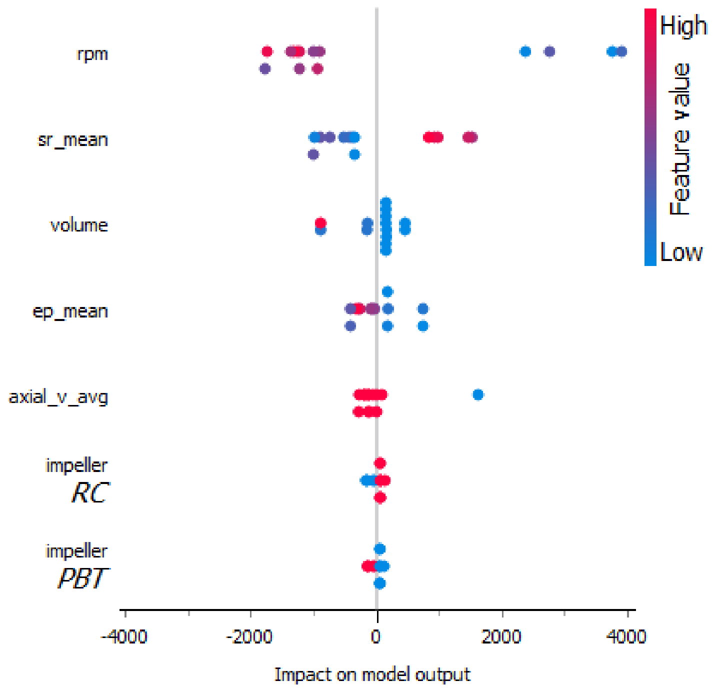

Figure 4 Model interpretability summary plot for model T9
